# Digitalized Clinical Data, Evidence and Transparency - Digitalization in Depression Treatment in the DECIDE Project

**DOI:** 10.1192/j.eurpsy.2023.1814

**Published:** 2023-07-19

**Authors:** H. F. Wiegand, N. Momtahen, E. Frenschkowski, V. Pantle, D. Riedinger, M. Pilz, T. Panholzer, A. Scherrer, K. Lieb

**Affiliations:** 1 Department of Psychiatry and Psychotherapy; 2 IMBEI, University Medicine of the Johannes Gutenberg University Mainz, Mainz; 3 Fraunhofer Institute for Industrial Mathematics ITWM, Kaiserslautern, Germany

## Abstract

**Introduction:**

Routine psychiatric treatment in Germany suffers from a lack of information exchange in a sectored care system, long and complex treatment courses, insufficient evidence orientation with increasingly complex clinical knowledge, a lack of qualified personnel, and lack of patient involvement. How can a digital solution counteract these problems?

**Objectives:**

First, discussion of problems in the care system and second, presentation of the concept and challenges of the DECIDE project, a Decentralized digital Environement for Consultation, data Integration, Decision making and patient Empowerment

**Methods:**

The project plan and first results will be presented of

1. surveys of patients and mental health professionals needs and concerns about digital solutions
2. focus groups
3. the software solution for mental health professionals and the connected app soltion for patients

**Results:**

Improtant functions of the DECIDE solution for mental health professionals should include:

- digitize clinical data to represent longitudinal treatment trajectories and plans and, in the face of increasingly complex knowledge, implement a transparent decision support system based on current guidelines for depression and important comorbidities in a disinterested way secure and transparent data exchange and access in practitioner networks involve patients in this exchange via app/web solution based on patient guidelines for transparency, empowerment and collaborative decision making review improved treatment algorithms in the long term using artificial intelligence based on digitized data lean data collection and compatibility with billing, appointment management, findings management and medication plan solutions.

**Conclusions:**

In psychiatry and psychotherapy digitization of clinical data for transparent exchange between practitioners and patients, presentation of progressions and treatment plans, and evidence-based decision support have a great potential. However, standardization, compatibility and collection of complex data remain a challenge.

**Disclosure of Interest:**

None Declared

